# In this month’s Bulletin

**DOI:** 10.2471/BLT.20.000220

**Published:** 2020-02-01

This month’s theme is universal health coverage, linked to the Prince Mahidol Award Conference in Bangkok, Thailand.

In the editorial section, Viroj Tangcharoensathein et al. (78) explain why it is time to deliver on political promises of extending health coverage to all people. Elizabeth Mason et al. (79) focus on the particular needs of women, children and adolescents. Susan P Sparkes and Joseph Kutzin (80) provide a reminder of the need to include HIV prevention and care services. 

In the news section, Gary Humphreys (83–84) reports on efforts to coordinate coverage over the geographical expanse of the Caribbean islands. Midori de Habich speaks to Gary Humphreys (85–86) about policies leading to universal health coverage in Peru.

## Australia, Cambodia, China, European Region, Fiji, Ghana, Kenya, Lebanon, Morocco, South Africa, Uganda, United States of America, Viet Nam, Zambia

### Educate and empower 

Giorgio Cometto et al. (109–116) discuss good practices in health workforce management.

## Brazil, Kenya, United Republic of Tanzania

### Designing the next phase of the HIV response 

Charles B Holmes et al. (87–94) weigh the pros and cons of targeted programmes.

## Chile, Columbia, Czechia, Gabon, Ghana, Germany, India, Indonesia, Japan, Kyrgyzstan, Mexico, Mongolia, Peru, Republic of Korea, Republic of Moldova, Rwanda, Spain, Switzerland, Thailand, Turkey, Ukraine, United Kingdom of Great Britain and Northern Ireland, Viet Nam

### Improving redistributive capacity

Inke Mathauer et al. (132–139) provide a global overview of pooling reforms.

## China, Ghana, India, Kyrgyzstan, Mongolia, South Africa, United States of America

### Overcoming the limitations of global targets

Sarah Louise Barber et al. (95–99) recommend costing exercises for national plans to increase coverage.

## Kenya

### Tracking funds

Ileana Vilcu et al. (126–131) link health information systems to strategic purchasing reforms.

## Senegal

### Improving community-based schemes

Bocar Mamadou Daff et al. (100–108) examine national financial protection measures.

## Thailand

### Trust through transparency

Kanang Kantamaturapoj et al. (117–125) review participatory approaches used to inform health financing policies.

### Leveraging domestic tax revenue

Viroj Tangcharoensathein et al. (140–145) dissect the political economy of an universal coverage scheme.

## Global

### Reaching every child

Mickey Chopra et al. (146–148) emphasize the need to achieve equitable immunization coverage.

